# Evaluation of PCR in Bronchoalveolar Lavage Fluid for Diagnosis of *Pneumocystis jirovecii* Pneumonia: A Bivariate Meta-Analysis and Systematic Review

**DOI:** 10.1371/journal.pone.0073099

**Published:** 2013-09-04

**Authors:** Li-Chao Fan, Hai-Wen Lu, Ke-Bin Cheng, Hui-Ping Li, Jin-Fu Xu

**Affiliations:** Department of Respiratory Medicine, Shanghai Pulmonary Hospital, Tongji University School of Medicine, Shanghai, China; California Department of Public Health, United States of America

## Abstract

**Background:**

As a promising tool, PCR in bronchoalveolar lavage fluid (BALF) has not been accepted as a diagnostic criterion for PJP.

**Objective:**

We undertook a systematic review of published studies to evaluate the diagnostic accuracy of PCR assays in BALF for PJP.

**Methods:**

Eligible studies from PubMed, Embase and Web of Science reporting PCR assays in BALF for diagnosing PJP were identified. A bivariate meta-analysis of the method’s sensitivity, specificity, and positive and negative likelihood ratios with a 95% confidence interval (CI) were analyzed. The post-test probability was performed to evaluate clinical usefulness. A summary receiver operating characteristics (SROC) curve was used to evaluate overall performance. Subgroup analyses were carried out to analysis the potential heterogeneity.

**Results:**

Sixteen studies published between 1994 and 2012 were included. The summary sensitivity and specificity values (95% CI) of PCR in BALF for diagnosis of PJP were 98.3% (91.3%–99.7%) and 91.0% (82.7%–95.5%), respectively. The positive and negative likelihood ratios were 10.894 (5.569–21.309) and 0.018 (0.003–0.099), respectively. In a setting of 20% prevalence of PJP, the probability of PJP would be over 3-fold if the BALF-PCR test was positive, and the probability of PJP would be less than 0.5% if it was negative. The area under the SROC curve was 0.98 (0.97–0.99).

**Conclusions:**

The method of PCR in BALF shows high sensitivity and good specificity for the diagnosis of PJP. However, clinical practice for the diagnosis of PJP should consider the consistent respiratory symptoms, radiographic changes and laboratory findings of the suspected patients.

## Introduction

Pneumocystis jirovecii pneumonia (PJP) remains an important cause of morbidity and mortality in all immunosuppressed patients especially in HIV-infected patients [1]–[3]. In England, laboratory-confirmed cases of *P. jirovecii* pneumonia had an average annual increase of 7% per year during 2000 to 2010 [4]. In the United States, the HIV Outpatient Study (HOPS) reported that the incidence of a first episode of PCP was 3.9 cases per 1,000 person-years for the period from 2003 to 2007 [5]. The mortality of in hospital PJP ranged from 7 to 11%. As for critical ill PJP patients, the mortality was as high as 29–62% [6].

Since *P. jirovecii* is hard to be cultured in vitro, the diagnosis of PJP relies on a direct microscopic examination of respiratory specimens. In non-HIV immunocompromised patients, conventional staining methods showed low sensitivity, ranging from 38% to 53% in sputum samples [7]. The microscopic approaches highly depend on quality and type of samples, the skill of observers and the reaction of cysts or trophozoites to the staining methods. Additionally, the diagnosis can be hampered in patients using highly active anti-retroviral therapy and PJP chemoprophylaxis which may lead to low burden of *P. jirovecii*, especially in sputum and oropharyngeal wash samples. Compared to sputum samples, positive results of BALF and lung biopsy specimens were considered as the“gold standard” for PJP diagnosis [8].

As a promising tool, PCR techniques for the diagnosis of PJP have been developed by many studies. However, the role of PCR assays in BALF remains controversial, especially to the patients with the positive PCR assays and the negative staining results. Thus, we systematically reviewed all relevant studies published from 1994 to 2012 to evaluate the diagnostic accuracy of PCR in BALF for the diagnosis of PJP.

## Materials and Methods

### Searching Strategy

A search was made of PubMed, Embase and Web of Science databases for the English-language literature published up to November 2012 by two investigators, respectively. The syntax for the searches was as follows: “Pneumocystis jirovecii Pneumonia” or “PJP” or “Pneumocystis Carinii Pneumonia” or “PCP” and “Polymerase Chain Reaction” or “PCR” and “bronchoalveolar lavage fluid” or “BALF”. An expanded hand-search of references of eligible trials and relevant articles were also performed.

### Inclusion Criteria

Full-text publications were included if: (1) They used PCR in bronchoalveolar lavage fluid for suspected PJP. (2) The diagnosis of PJP had to be made in patients who had (i) relevant clinical manifestations including cough and fever; (ii) detection of the pathogen in lung tissue, BALF specimens or sputum specimens by use of conventional staining methods (Giemsa, Toluidine-blue and Gomori methenamine silver) or indirect immunofluorescence assay; (iii) radiographic findings on chest X-ray and chest computed tomography (CT) compatible with PCP. (3) Studies reported that the PCR diagnostic data that could not make mathematical sense according to known mathematical formulas were excluded.(4)To avoid bias, studies with less than 10 patients suspicious of PJP were excluded. Disagreements were discussed among the group until a consensus was reached.

### Quality Assessment

Each article that met eligible criteria was assessed using the QUADAS-2 [9] tool by two investigators, respectively. Discrepancies were solved by consensus, and there was an adjudicator in case of persistent disagreement.

### Publication Bias

Deek’s funnel plot asymmetry test was performed to detect publication bias [10].

### Data Extraction

Data extraction was done independently by two reviewers with predesigned data extraction forms including the following items: general identification information (patient population, study design, reference standard, blind and proven PJP/total patients), technical details of the PCR methods (BALF sample volume, sample centrifugation, DNA extraction methods, PCR method, target gene and appropriate control). Data was directly extracted from the published data, or estimated using common mathematical formulas.

### Statistical Analysis

We calculated pooled estimates of the sensitivity (SEN), specificity (SPE) by using a bivariate analysis approach [11]. The positive and negative likelihood ratio (PLR and NLR) and the diagnostic odds ratio (DOR) were also reported. We constructed a summary receiver operating characteristics (SROC) curve to see the accuracy of the assay.

Statistical heterogeneity of the results of the trials was assessed by the chi-square test, expressed with the I^2^ index, as described by Higgins and his colleagues [12]. When heterogeneity was detected, threshold analysis and multiple covariates bivariate meta-regression models were performed [13]. The main covariates assessed were sample processing (prospective), study design (cohort), DNA extraction method (commercial), type of PCR (qPCR; single-PCR; nested-PCR), target gene (mt-rRNA; DHPS), staining methods (IFS), BALF centrifugation and blind. Forest plots were created for each study, showing individual outcome with confidence intervals (CI) and the overall DerSimmonian-Laird pooled estimate [14]. All P values were two-sided and P<0.05 were considered statistically significant. Statistical analyses were performed using midas module in Stata software v. 12 (Stata Corporation, College Station, TX).

## Results

### Trial Flow

**Figure 1:**
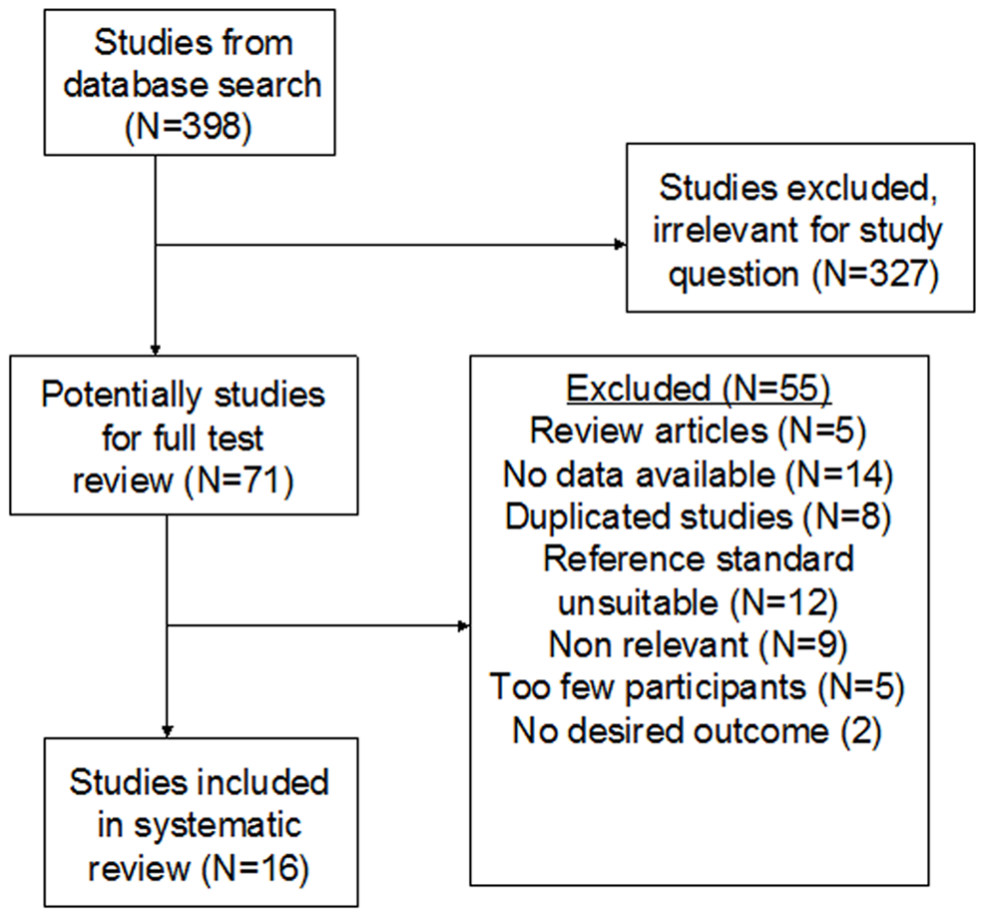
Flow diagram of the process of selecting included studies. This flow-diagram shows 398 references identified, after screening all of the titles and abstracts, 71 articles were selected for full-text review. Ultimately, 16 studies that were included in the meta-analysis, 55 studies were excluded for various reasons.

An initial search identified 398 potentially relevant studies. After reading the titles and abstracts, 327 studies were excluded because of irrelevance. With further screening of full texts, 55 studies were discarded for various reasons. Ultimately, 16 studies with 1857 BALF samples from 1793 patients published between 1994 and 2012 met the inclusion criteria and were included in the systematic review [15]–[30]. The literature screening process was shown in Figure 1.

### Characteristics of Eligible Trials

The characteristics of the included studies were summarized in Table 1. We included prospective, retrospective, cohort and case-control studies. The populations evaluated in our meta-analysis were consisted of HIV-infected patients and non-HIV immunosuppressed patients who were suspected of having PJP. The criteria used for diagnosis of PJP were almost based on chest radiographic infiltration and clinical presentation along with etiological findings from respiratory samples. Details of the PCR techniques were presented in Table 2.

**Table 1:** Characteristics of the included studies.

Study reference no.	Patient population	Study design	Staining Method (s)	Radiology-based	Clinical Presentations-based	Blind	Proven PJP/total patients
15	HIV, SOC, HM, SLE, OT	Retrospective cohort	CS, IFS	Y	Y	Y	54/102
16	Non-AIDS	Retrospective case-control	CS	Y	Y	NR	20/60
17	HIV, OT, CA,	Retrospective cohort	CS	Y	Y	NR	21/143
18	HM, OT, SOC, BMT, CD	Retrospective cohort	IFS	Y	Y	Y	27/351
19	HIV	Retrospective case-control	CS	Y	Y	Y	61/132
20	HIV	Prospective cohort	CS	NR	Y	Y	10/17
21	HIV, BMT, HM, CD, OT, WG	Retrospective cohort	CS, IFS	NR	NR	Y	41/124
22	HIV, HM, SOC	Retrospective case-control	CS	Y	Y	Y	71/141
23	HIV, OD	Prospective cohort	CS	Y	Y	NR	12/21
24	HIV	Retrospective case-control	CS	Y	Y	NR	71/146
25	HIV	Prospective case-control	CS, IFS	NR	Y	Y	28/61
26	HIV, HM	Prospective case-control	CS	Y	Y	NR	57/132
27	HIV	Retrospective case-control	CS	NR	Y	Y	40/123
28	HIV	Prospective cohort	IFS	Y	Y	Y	20/59
29	HIV	Retrospective cohort	IFS	N	Y	NR	23/61
30	HIV	Retrospective case-control	CS	Y	Y	NR	50/120

Abbreviations: BMT, bone marrow transplants; CA, cancer; CD, corticosteroid dependent; CS, colorimeric staining; CA, cancer; HIV, hunman immunodeficiency virus; HM, haematological malignancies; IFS, immunofluorescent staining; Non-AIDS, non-acquired immune deficiency syndrome; NR, not reported; OD, other diseases; OT, organ transplantation; SOC, solid organ malignancies; SLE, systemic lupus erythematosus; WG, wegener’s granulomatosis; Y, yes.

**Table 2:** Technical details methods of the PCR in the included studies.

Studyreference no.	BALF samplevolume(ml)	Samlecentrifugation	DAN extractionmethods	PCR method	target gene	Appropriatecontrol
15	3–5	Y	QIAamp	nested PCR	mt LSU rRNA	Y
16	0.2	Y	proteinase	conventional PCR	mt LSU rRNA	Y
17	0.5	Y	proteinase	qPCR	Kex-1	Y
18	NR	Y	QIAamp	single PCR	mt rRNA	Y
19	0.2; 0.75	NR	DNeasy, QIAamp	conventional PCR	mt LSU rRNA	Y
20	NR	Y	Wizard purification	RT-PCR	mt LSU rRNA	Y
21	0.2	Y	Qiagen	real-time PCR	DHPS	NR
22	1	NR	QIAamp	real-time PCR	DHPS	Y
23	NR	Y	phenol chloroform	single PCR	5S rRNA	Y
24	0.25	NR	phenol chloroform	nested PCR	ITS	Y
25	2	Y	phenol-chloroform/Chelex	TD-PCR	mt LSU rRNA	Y
26	NR	Y	QIA purification	nested PCR	mt LSU rRNA	Y
27	0.1	Y	proteinase K	nested PCR	mt rRNA	Y
28	NR	NR	proteinase K	single PCR	mt LSU rRNA	Y
29	1	NR	phenol-chloroform	nested PCR	mt LSU rRNA	Y
30	10	Y	phenol-chloroform	single PCR	mt rRNA	NR

Abbreviations: DHPS, dihydroperoate synthase; ITS, internal transcribed spacer; mt LSU rRNA, mitochondrial large-subunit ribosomal RNA; mt rRNA, mitochondrial ribosomal RNA; NR, not reported; qPCR, quantitative polymerase chain reaction; RT-PCR, reverse transcriptase -polymerase chain reaction; TD-PCR, a single-round touchdown PCR; Y, yes.

### Validity of Included Trials

**Figure 2:**
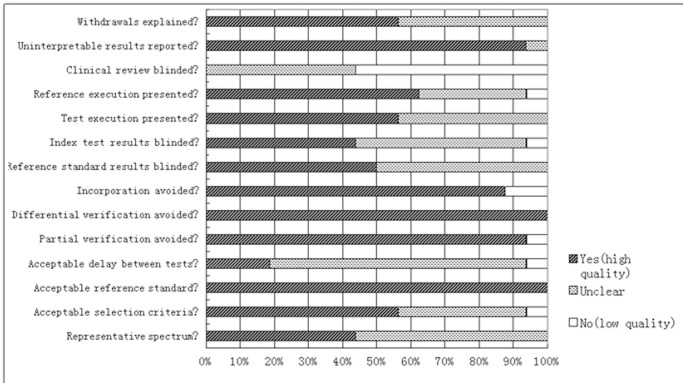
Summary of the methodological quality assessment of the included studies according to QUADAS-2 criteria. Data was carried out using a bar graph showing the percentages of the 16 studies that met the criteria (Yes), did not meet the criteria (No), and did not provide adequate relevant data (Unclear).

Methodology quality of included trials was presented in Figure 2. Based on the methods reported in each trial, each of the 14 components according to QUADAS-2 criteria was graded “yes”, “unclear” or “no”, which meant “low risk of bias”, “uncertain of bias” and “high risk of bias”, respectively.

Non-publication bias was detected by Deek’s funnel plot asymmetry test (P = 0.671).

### Data Synthesis and Meta-analysis

**Figure 3:**
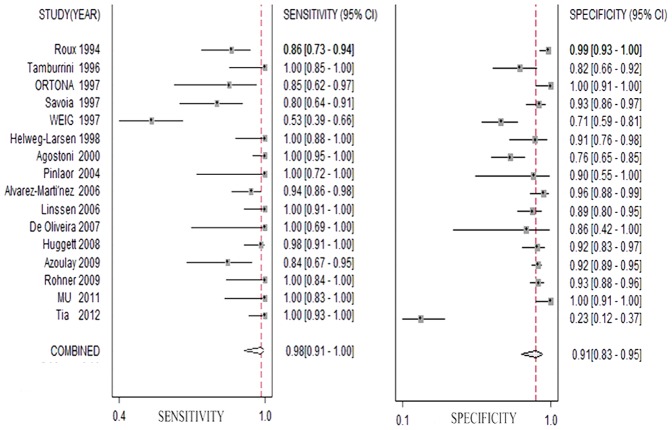
Forest plots of the sensitivity and specificity of BALF-PCR for the diagnosis of PJP. The circles in squares and the horizontal lines represent the point estimate and 95% confidence interval for each included study and the diamond represents the pooled estimate.

**Figure 4:**
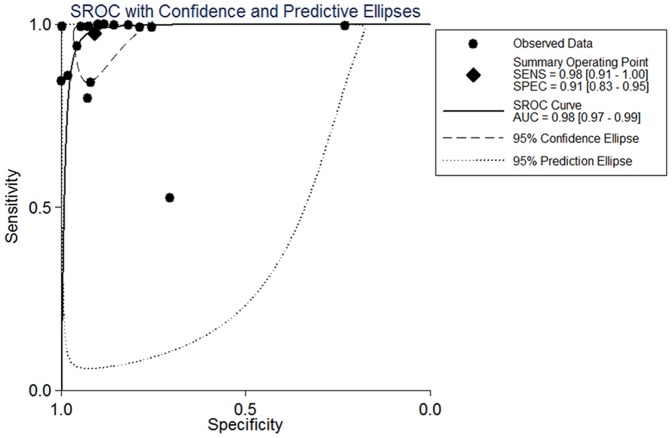
SROC curve shows summary operating sensitivity and specificity with confidence and prediction contours. SROC curve with confidence and prediction regions around mean operating sensitivity and specificity point analyses of PCR assays in BALF for the diagnosis of PJP. Abbreviations: AUC, area under curve; SENS, sensitivity; SPEC, specificity; SROC curve, summary receiver operating (SROC) curve.

Forest plots of sensitivity and specificity showed each study and the overall studies. For 16 included studies, the sensitivity values ranged from 53% to 100% and the specificity values ranged from 23% to 100%, respectively. The pooled sensitivity and specificity values of the bivariate model were 98.3% (95% CI, 91.3% to 99.7%) and 91.0% (95% CI, 82.7% to 95.5%), respectively (Figure 3). The average positive likelihood ratio of PCR in BALF was 10.894 (95% CI, 5.569–21.309) and the negative likelihood ratio was 0.018 (95% CI, 0.003–0.099). Based on the positive and negative likelihood ratios obtained from the meta-analysis, the interpretations of the BALF-PCR results for the different pretest probabilities could be examined by using Bayes theorem to generate post-test probabilities [31], PTP = LR×pretest probability/[(1-pretest probability)×(1-LR)]. For instance, in a setting of 20% prevalence of PJP, the probability of PJP would be less than 0.5% if the BALF-PCR test was negative and the probability of PJP would be more than 70% if the BALF-PCR test was positive. The pooled diagnostic odds ratio (DOR) was 595.670 (95% CI, 106.412–3334.420) and the area under the curve (AUC) was 0.98 (95% CI, 0.97–0.99) (Figure 4), indicating that the PCR assay has an excellent level of diagnostic value for PJP. We found significant heterogeneity for all test performances because I-square values were above 50%. In subgroup analyses, the sensitivity of subgroups were all above 90% and the specificity were all above 80%. And except cohort, comEX and nested-PCR subgroups, the SEN and SPE of the remaining ones were all over 90%. Quantitative PCR had the highest SEN of 100% (95% CI, 92%–100%) and SPE of 93% (95% CI, 77%–98%). The SEN of single-PCR was relatively low, 92% (95% CI, 54%–99%), while the SPE was 97% (95% CI, 88%–99%). In the analysis of potential influence of heterogeneity, I-squared of comEX, nested-PCR and single PCR subgroups were over 50%. However, we did not find statistically significant except nested-PCR (P<0.001) (Table 3).

**Table 3:** Subgroup analyses of PCR in BAL fluid for the diagnosis of PJP.

Subgroup study	Sensitivity	Specificity	I-squared(95% CI )	LRT Chi	P value
prospective	0.96 [0.66–1.00]	0.91 [0.71–0.98]	0.00 [0.00–100.00]	0.61	0.74
mt-rRNA	0.96 [0.83–0.99]	0.91 [0.80–0.96]	43.38 [0.00–100.00]	3.53	0.17
cohort	0.99 [0.91–1.00]	0.88 [0.72–0.95]	0.00 [0.00–100.00]	1.86	0.40
comEX	0.98 [0.82–1.00]	0.84 [0.66–0.93]	52.83 [0.00–100.00]	4.24	0.12
nested-PCR *	0.98 [0.76–1.00]	0.73 [0.53–0.86]	82.19 [62.16–100.00]	11.23	0.00
qPCR	1.00 [0.92–1.00]	0.93 [0.77–0.98]	22.41 [0.00–100.00]	2.58	0.28
blind	0.98 [0.88–1.00]	0.91 [0.78–0.96]	0.00 [0.00–100.00]	0.09	0.96
centrifugation	0.98 [0.86–1.00]	0.90 [0.79–0.96]	0.00 [0.00–100.00]	0.41	0.81
single-PCR	0.92 [0.54–0.99]	0.97 [0.88–0.99]	57.37 [3.85–100.00]	4.69	0.10
DHPS gene	0.99 [0.66–1.00]	0.94 [0.67–0.99]	0.00 [0.00–100.00]	0.31	0.86
IFS	0.98 [0.70–1.00]	0.94 [0.78–0.98]	0.00 [0.00–100.00]	0.42	0.81

The use of regression methods to incorporate the effect of covarying factors on summary measures of performance to explore between-study heterogeneity. We did not find statistically significant except nested-PCR * (P<0.001). Abbreviations: 95% CI, 95% confidence interval; comEX, commercial extraction; DHPS, dihydroperoate synthase; IFS, immunofluorescent staining; LRT Chi, likelihood ratio Chi; mt-rRNA, mitochondrial ribosomal RNA; qPCR, quantitative polymerase chain reaction.

## Discussion

Despite the introduction of highly active antiretroviral therapy (HAART), Pneumocystis jirovecii still remains a severe opportunistic infection, associated with a high mortality rate. Recently, a paper reported that the proportion of PCP as cause of death significantly increased from 8.7% in pre-HAART (1990–1997) to 31.8% in late-HAART (2002–2011) period (P<0.001) [32]. At present, a highly sensitive technique is required for the detection of Pneumocystis jirovecii, especially in patients receiving chemoprophylaxis and aggressive anti-retroviral therapy, because false negative results by tinctorial methods may occur in these patients owing to the low sensitivity of these assays.

We undertook the meta-analysis aiming to evaluate the overall accuracy of the BALF-PCR for the diagnosis of PJP. By meta-analysis, the pooled sensitivity of the BALF-PCR for the diagnosis of PJP was 98.3% (95% CI, 91.3%–99.7%) and the pooled specificity was 91.0% (95% CI, 82.7%–95.5%), which predicts PCR in BALF is a very good method for diagnosis of PJP.

We also investigated the causes of heterogeneity. For the whole population, there was a significant heterogeneity due to a threshold effect (p = 0.7969). In addition, we could not extract the exact data for AIDS and non-AIDS patients and the number of patients that were treated with prophylaxis prior to BALF, which maybe the potential causes lead to heterogeneity. Subgroup analysis showed nested PCR had a relatively high sensitivity of 98% (95% CI, 76%–100%) and the lowest specificity of 73% (95% CI, 53%–86%), which was consistent with Moonens’s finding [33]. Meta-regression showed the I-squared (95% CI) of nested-PCR was 82.19 (62.16–100.00) with p<0.001, indicating a significant influence of heterogeneity. Another potential influenced factor was the lack of a universally accepted definition for the diagnosis of PCP. The QUADAS-2 reference standard risk of bias similarly affected results. Results were heterogeneous, and the paucity of studies did not allow for assessment of some covariates.

**Figure 5:**
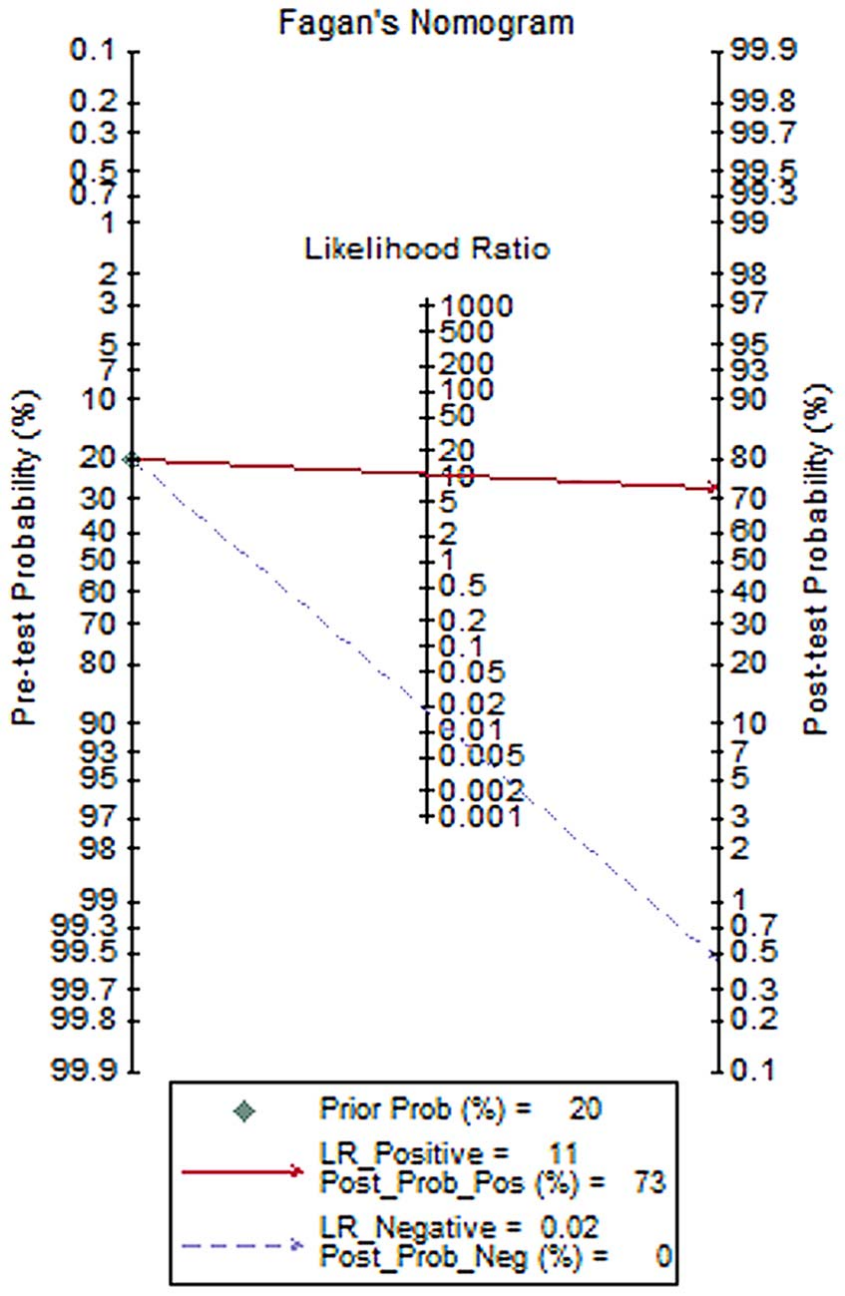
Fagan’s nomogram for calculating post-test probabilities (PTPs). Lines were drawn from the prior probability on the left through the likelihood ratios in the center and extended to the posterior probabilities on the right, which demonstrates that BALF-PCR is very informative raising probability of PJP to over 3-fold when positive from 20% and lowering the probability of disease to as low as 0.5% when negative.

In clinical practice, whether BALF-PCR is appropriate as a diagnostic test depends ultimately on the predictive values in the intended setting. Fagan’s nomogram showed that in a setting of 20% prevalence of PJP, the probability of PJP would be less than 0.5% if the BALF-PCR test was negative and the probability of PJP would be more than 70% if the BALF-PCR test was positive, which is very helpful for the diagnosis of PJP (Figure 5). Especially in the pre-HAART era, fibrotic bronchoscopy with BALF should be performed in patients with negative induced sputum results while presenting compatible clinical symptoms.

*P. jirovecii*-specific DNA amplification will be helpful if the results of routine microbiological methods are negative to patients clinically suspected with PJP. Discrepancies between positive PCR and negative staining results have always been reported by many investigators. Since neither calcofluor white stain [34] nor the monoclonal antibody (fluorescent) stains [35] can make a definitive identification for PJP, positive PCR results would provide an earlier diagnosis for suspected patients before morphological methods. Lu et al. reviewed the PCR methods for diagnosis of PJP. They found that 31.8% of the patients with current false-positive PCR results had prior or later PJP [36]. Other studies made the similar conclusion [7], [37], which suggesting that a possible explanation for the differences in assays’ sensitivity may be caused by the low load of *P. jirovecii*. In this situation, repeating the staining assay from BALF along with re-evaluating consistent respiratory symptoms and radiographic changes for the diagnosis of PJP are recommended.

It should be noted that *P. jirovecii* DNA has been detected in respiratory samples from patients either with colonization or subclinical infection due to the high sensitivity of the molecular methods [38], [39]. Quantification of the Pneumocystis jirovecii burden by real-time PCR might be helpful to distinguish between colonization and infection and could possibly allow for therapeutic monitoring. Matsumura et al. found that the area under the receiver-operating characteristic curve (AUC), sensitivity and specificity for discriminating definite PJP from colonization were 0.96, 100.0% and 80.0%, respectively, at a cut-off value of 1300 copies/ml [40]. However, Flori P et al. found an overlap cut-off values between in samples obtained from potential carriers and proven PJP [41]. Due to the performance of different in-house PCR assays currently used in microbiology laboratories, the establishment of a uniform PCR assay and a cut-off value to discriminate between disease and colonization for *P. jirovecii* is utmost importance.

Our study had some limitations. First, the overall number of patients included in our review was relatively small. Albeit we tried to collect all relevant data, it was hard to ensure that no data was missed. Second, we ruled out non-English-language studies and studies with less than 10 cases, which may lead to bias. Third, the lack of a published gold standard for the diagnosis of PJP may cause underlying heterogeneity in our study. Although we try to include studies using criteria that patients who had relevant clinical and radiological manifestations with detection of the pathogen in lower respiratory secretions, we found that some studies did not adhere to this criteria rigorously.

In summary, this meta-analysis shows a very high accuracy of PCR in BALF for the diagnosis of PJP to patients at risk. The excellent sensitivity of PCR for PJP in BALF suggests that a negative result in suspected patients should present ruling out the diagnosis of PJP. A positive PCR result should be interpreted in parallel with compatible clinical and radiological findings. Further prospective cohort studies should focus on quantitative PCR standardization and determination of the optimal cut-off for quantitative PCR results for the wide use of this test in clinical practice.

## Supporting Information

Checklist S1. (DOC)
